# “Redo” 2D–3D Fusion Technique during Endovascular Redo Aortic Repair

**DOI:** 10.3390/diagnostics13040635

**Published:** 2023-02-08

**Authors:** Fabrizio Minelli, Simona Sica, Fadia Salman, Federica Donato, May Dvir, Yamume Tshomba, Giovanni Tinelli

**Affiliations:** 1 Unit of Vascular Surgery, Fondazione Policlinico Universitario Gemelli IRCCS, Roma—Università Cattolica del Sacro Cuore, 00168 Rome, Italy; 2 School of Medicine and Surgery, Università Cattolica del Sacro Cuore, 00168 Rome, Italy

**Keywords:** endovascular procedures, redo aortic endovascular surgery, fusion imaging, redo fusion imaging, personalized medicine

## Abstract

Purpose: The present study aims to describe a new 2D–3D fusion registration method in the case of endovascular redo aortic repair and compare the accuracy of the registration using the previously implanted devices vs. bones as landmarks. Materials and Methods: This single-center study prospectively analyzed all the patients that underwent elective endovascular re-interventions using the Redo Fusion technique between January 2016 and December 2021 at the Vascular Surgery Unit of the Fondazione Policlinico Universitario A. Gemelli (FPUG)—IRCCS in Rome, Italy. The fusion overlay was performed twice, first using bone landmarks (bone fusion) and the second using radiopaque markers of a previous endovascular device (redo fusion). The pre-operative 3D model was fused with live fluoroscopy to create a roadmap. Longitudinal distances between the inferior margin of the target vessel in live fluoroscopy and the inferior margin of the target vessel in bone fusion and redo fusion were measured. Results: This single-center study prospectively analyzed 20 patients. There were 15 men and five women, with a median age of 69.7 (IQR 42) years. The median distance between the inferior margin of the target vessel ostium in digital subtraction angiography and the inferior margin of the target vessel ostium in bone fusion and redo fusion was 5.35 mm and 1.35 mm, respectively (*p* ≤ 0.0001). Conclusions: The redo fusion technique is accurate and allows the optimization of X-ray working views, supporting the endovascular navigation and vessel catheterization in case of endovascular redo aortic repair.

## 1. Introduction

The use of endovascular techniques has increased over the last decade. The evolution of device technology has allowed physicians to perform more complex, minimally invasive aortic endovascular repairs. Imaging systems have also evolved to facilitate these challenging procedures [1]. The latest generation of hybrid operating rooms (HOR) is equipped with advanced imaging tools, such as fusion imaging (FI), associated with better procedural and short-term outcomes and potential clinical long-term benefits [2]. The use of image fusion guidance has been associated with several procedural benefits. Prior publications have reported a significant reduction in both operator and patient radiation exposure and contrast medium injection with the routine use of fusion guidance by trained operators applying the ‘as low as reasonably achievable’ (ALARA) principles [3,4]. 

Fusion imaging is a technique that allows three-dimensional (3D) visualization of intraoperative landmarks by projecting 3D images derived from the pre-operative computed tomography (CT) angiography (CTA) scan onto a two-dimensional (2D) intraoperative fluoroscopic image (2D–3D fusion imaging) [5]. Registration can be either 2D–3D, which is performed by superimposing the 3D bone model obtained from the CTA onto the bony structures on 2D fluoroscopic images (which requires two perpendicular 2D images), or 3D–3D by superimposing the CTA 3D bone model and aortic calcifications on to a 3D bone model obtained from an on-table cone-beam CT. However, performing a CBCT at the beginning of the procedure can at times be logistically challenging and is associated with significant radiation exposure [6]. 

**Figure 1:**
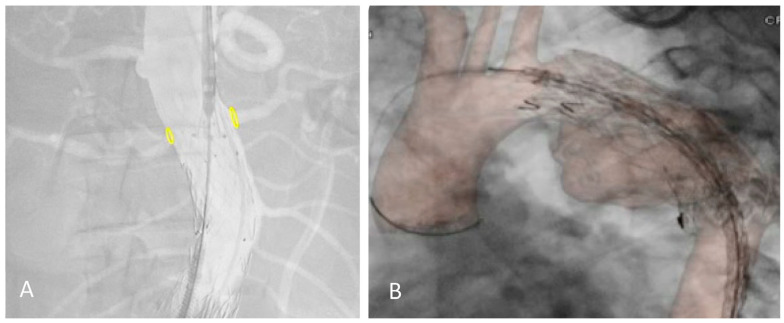
Proximal relining for type IA endoleak in a previous EVAR (**A**) and TEVAR (**B**) under the redo fusion technique. Accuracy of the yellow markers on bilateral renal arteries after shaft device insertion and in the thoracic aorta.

The 2D–3D FI technique relies on bones as anatomical landmarks. However, the pre-operative CTA is performed with the upper limbs in hyper-abduction, creating a potential mismatch with the intraoperative position of the patient (arms adducted with the elbows extended). A discrepancy between our fusion reconstruction and the real target vessels position can be observed, especially after stiff guide and shaft device insertion, and in the thorax overlay (Figure 1).

Patients who undergo endovascular aortic repair remain at risk for re-intervention indefinitely. In the three years after endovascular aneurysm repair (EVAR), up to one-fifth of patients require a re-intervention procedure [7]. Re-interventions after EVAR, thoracic endovascular aneurysm repair (TEVAR) or complex fenestrated (F-) or branched (B-) EVAR are frequently treated with different endovascular strategies, which together comprise endovascular redo aortic repair. Redo aortic operations are technically demanding and are carried out with increased risks. Improving technology, such as fusion imaging, should mitigate some of this risk [8]. 

In case of patients with previous endovascular devices such as stents, vascular plugs, or endoprosthesis, it is possible to obtain a fusion image with the previously implanted devices as landmarks. 

The present study aims to describe a new 2D–3D fusion registration method in the case of endovascular redo aortic repair and compare the accuracy of the registration using the previously implanted devices (redo fusion) vs. bones (bone fusion) as landmarks.

## 2. Materials and Methods

This single-center study prospectively analyzed all the patients that underwent elective endovascular re-interventions using the redo fusion technique between January 2016 and December 2021 at the Vascular Surgery Unit of the Fondazione Policlinico Universitario A. Gemelli (FPUG)—IRCCS in Rome, Italy. 

Indications for re-interventions included: type I and II endoleaks; staged procedures for thoracic, thoraco-abdominal and complex abdominal aortic aneurysms; and chronic type B dissection treatment. Emergency procedures or procedures conducted without fusion imaging were excluded.

All of the procedures were performed in an HOR (Artis Zeego; Siemens Healthcare GmbH, Forchheim, Germany), under general anesthesia, by a single experienced operator. Imaging analysis and collection of measurement data were performed independently by two vascular surgeons experienced in aortic graft planning according to the standardized protocol. The two observers used the same standardized measurement technique, and each observer repeated all of the measurements two times for each patient.

*Bone Fusion Technique*

Before each procedure, bone and aortic 3D models were reconstructed from the preoperative CTA scan (performed within three months prior to the procedure) on a workstation (Leonardo, Healthcare Sector, Siemens AG, Forchheim, Germany) and sent to the X-ray system. The pre-operative CTA was processed by placing digital marks around the ostium of the target vessels on the CTA. It was then fused with live fluoroscopy. Registration of this 3D preoperative model was performed using bone landmarks visible on two fluoroscopic orthogonal shots (anterior–posterior and lateral) of the spine. During the procedure, this layout was used to center the region of interest, and to adjust collimation, without requiring fluoroscopy. The position of the target vessels was confirmed by a 6-cc contrast medium injection at 30 cc/s performed after the stiff wire insertion.

*Redo Fusion Technique*

**Figure 2:**
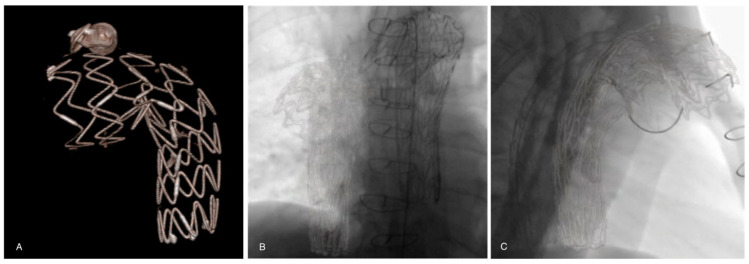
(**A**) A previous thoracic stent-graft three-dimensional volume rendering model reconstructed from the preoperative CTA. Registration and overlay are performed with two fluoroscopic orthogonal antero-posterior (**B**) and lateral (**C**) shots.

Before each procedure, aortic 3D models were reconstructed from the pre-operative CTA scan (performed within three months prior to the procedure) on a workstation (Leonardo, Healthcare Sector, Siemens AG, Forchheim, Germany) and sent to the X-ray system. The pre-operative CTA was processed by placing digital marks around the ostium of the target vessels on the CTA. It was then fused with live fluoroscopy. Registration of this 3D pre-operative model was performed using stent grafts, vascular plugs, coils, or other radiomarkers from a previous endovascular procedure visible on two fluoroscopic orthogonal shots (anterior–posterior and lateral) of the spine (Figure 2). During the procedure, this layout was used to center the region of interest, and to adjust collimation, without the need for fluoroscopy. The position of the target vessels was confirmed by a 6-cc contrast medium injection at 30 cc/s performed after the stiff wire insertion.

*Bone Fusion vs. Redo Fusion comparison*

In the present study the overlay was performed twice: the first using bone landmarks (bone fusion), and the second using radiopaque markers of previous endovascular materials implanted in the aorta (redo fusion). Comparison of the accuracy of the alignment of the two techniques was performed by two operators with measurements taken twice for each technique.

All the definitions of the variables used are reported in Table 1. Longitudinal distances between the inferior margin of the ostium of the target vessel in live fluoroscopy and the inferior margin of the target vessel in bone fusion and redo fusion were measured. Intra-observer and interobserver variability, L1 and L2 distances, L1 and L2 measurements in two subgroups of patients with the predefined distance between fusion marker and target vessel (cut-off 3 cm), and L1 and L2 measurements in the two subgroups of patients with predefined aortic angle (cut-off 45°) were performed. Intra-observer variability was defined as the difference in repeated measurements by the same observer. Interobserver variability was defined as the difference in the measurements between the two observers. 

*Statistical analysis*

Continuous data are reported as the mean ± standard deviation (SD) or, in the case of Gaussian distribution, as the median with interquartile range (IQR). Two-group comparisons were performed with the paired *t*-test. The threshold for statistical significance was set at a *p*-value < 0.05. SPSS software (version 23; IBM Corporation, Armonk, NY, USA) was used for statistical analysis.

## 3. Results

This single-center study prospectively analyzed 20 patients who underwent elective endovascular re-interventions between January 2016 and April 2021 at the Vascular Surgery Unit of the FPUG in Rome, Italy. There were 15 men and five women, with a median age of 69.7 (IQR 42) years. Indications for re-interventions were type IA (*n* = 3) and II (*n* = 1) endoleaks, staged procedures for thoracic (*n* = 4), thoraco-abdominal (*n* = 7) and complex abdominal (*n* = 3) aortic aneurysms, and chronic type B dissection (*n* = 2) treatment. The patients’ details are reported in Table 2.

Table 3 shows the reproducibility of repeated distance measurements. The median difference of the intra-observer measurements was 0.85 mm and 0.81 mm for bone fusion and redo fusion, respectively (*p* = 0.2). The interobserver variation was 0.7 mm and 0.65 mm for bone fusion and redo fusion, respectively (*p* = 0.8).

The distance between the inferior margin of the target vessel ostium in DSA and the inferior margin of the target vessel ostium in bone fusion (L1) and redo fusion (L2) were 5.35 mm and 1.35 mm, respectively (*p* ≤ 0.0001) (Table 4).

To confirm the accuracy of the redo fusion method, the measurements were performed on two subgroups of patients (Table 5). The criteria were established based on the distance between the fusion marker and the target vessel (> or ≤3 cm). The measurements were statistically significant for the first subgroup (>3 cm) of seven patients (*p* < 0.0008), with an average distance of 5.28 mm and 1.60 in group L1 and L2, respectively. The second subgroup (≤3 cm) of 13 patients had an average distance of 5.38 mm and 1.21 mm in the L1 and L2 group, respectively (*p* < 0.0001).

Further measurements were performed on two other subgroups of patients, categorized based on an aortic angle of 45° at the level of new working area level (Table 6). The first subgroup of five patients (<45°) had an average distance of 4.40 mm and 1.40 mm in the L1 and L2 group, respectively (*p* < 0.01). The second subgroup of 15 patients (≥45°) had an average distance of 5.80 mm and 1.33 mm in the L1 and L2 group, respectively (*p* < 0.0001). 

## 4. Discussion

Over the decades, the development of endovascular aortic repair has revolutionized the treatment of different aortic pathologies. Endovascular repair has also gained widespread acceptance for thoraco-abdominal aortic diseases with F-BEVAR in a staged approach [9]. Endovascular repair is associated with lower peri-operative morbidity, mortality, and a shorter hospital stay when compared with open surgical repair. However, the primary tradeoff between endovascular and open surgery is the durability of the repair. Endovascular operations are associated with higher rates of re-intervention and late aneurysm rupture than open surgical repair [10]. A recent study from Columbo et al. on 12,911 patients reported a cumulative rate of re-intervention of 15% at three years and 33% at 10 years after EVAR [11]. The rates of re-intervention after TEVAR ranges from 10–15% for thoracic aortic aneurysms to 46% after complicated type B thoracic aortic dissections [12,13].

Risk factors for failure following aortic repair include larger abdominal aortic aneurysm in necks, severe neck angulation, as well as clinical variables [8]. Endovascular aortic repair is challenging, and redo aortic operations are technically demanding and are carried out with increased risks [8,14]. Most re-interventions are performed because of endoleak, stent migration, graft kinking with stenosis or occlusion, and proximal or distal disease progression [15]. Previous studies on aortic neck enlargement after endovascular repair of thoracic and infrarenal aortic aneurysms have found enlargement after endograft repair that could lead to device migration, endoleak, and rupture [16,17,18]. Moreover, many failed endovascular repairs could have been avoided if meticulous and accurate planning (with the significant contribution of pre-operative planning using 3D workstations) had been undertaken with an assessment of the potential for disease progression and endoprosthesis design [19]. In addition, suboptimal sealing is a significant determinant of endovascular treatment failure, whether due to severe angulation of the aorta or the presence of thrombi. All these factors should be considered in the initial planning of a 3D workstation [20,21].

With the ever-expanding applications of endovascular intervention, cases are growing in both quantity and complexity [22]. More complex cases inevitably lead to longer fluoroscopy time, more frequent DSA acquisitions, and greater radiation exposure to the patient and operator. Technologic innovation has played a key role in the evolution of the endovascular equipment during aortic repair. Several studies have reported the benefits of hybrid rooms reducing operators and patient radiation exposure, contrast medium volume or procedure time in standard or complex endovascular aortic repair [1,23,24,25].

**Figure 3:**
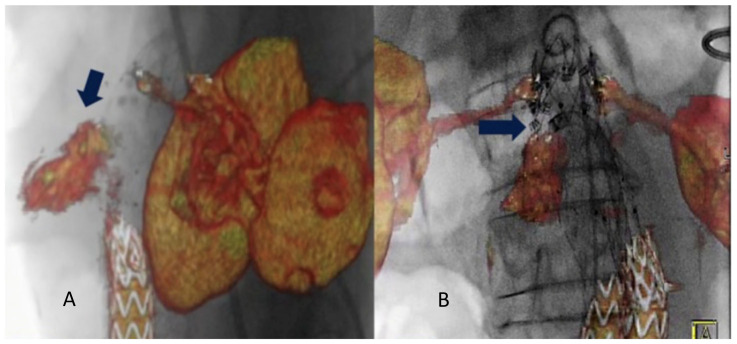
Intraoperative projection of type IA endoleak channel (**A**). Positioning and deployment of the Heli-FX endo-anchors in the specific origin of the endoleak (arrow) for the “endosuture line” (**B**) from Tinelli et al. [31].

Other frequently reported benefits of hybrid rooms are the higher image quality and the available advanced imaging applications such as fusion imaging, which eases navigation and increases the accuracy of endografts deployment [2]. Fusion imaging provides a continuous display of the vascular structures without the need for fluoroscopy. This allows positioning of the gantry and table, choice of working C-arm angulations, and adjustment of collimation and magnification without x-ray use. This technique has significantly reduced contrast agent volume, radiation exposure, and procedure time compared with a standard endovascular repair procedure [1,26]. Two different registration methods can be used to obtain fusion images: the 2D–3D method and the 3D–3D method. The 3D–3D method has been shown to reduce the rate of secondary operations and their associated morbidity [27]. Chinnadurai et al. reported a higher accuracy using aortic wall calcifications-based vs spine-based image fusion during 3D–3D fusion for EVAR guidance [28]. However, Schulz et al. [5] have reported that the 3D–3D method requires a higher radiation dose and contrast agent injection. Our study obtained fusion registration with only two orthogonal fluoroscopic shots. This method is almost radiation-free. Routine use of this technology has contributed to increased short- and long-term technical success rates of all endovascular procedures, especially F-BEVAR [29]. Tenorio et al. observed a higher technical success rate when F-BEVAR was performed in a modern hybrid room with fusion imaging and CBCT and lower rates of 30-day mortality and early re-interventions [30]. In case of endovascular redo aortic repair (staged procedures or re-interventions), it is possible to use the redo fusion technique. As described, this technique uses the previously implanted devices as landmarks for the FI overlay. In the literature, only one case of type IA endoleak treatment with endo-anchors deployment under redo fusion guidance is reported [31] Figure 3.

The present study established how, in real-time fusion imaging, the distance between the target vessel in 3D volume rendering and 2D fluoroscopy is effectively reduced by using endovascular markers to overlay the images. This is due to the proximity of the previously implanted marker (stent-graft, plugs, etc.) to the repair area, based on the intuitive concept that the closer the markers area, the lower the error rate of the fusion technique. At the same time, there is less use of contrast, also because the scaffold of the previously implanted prosthesis has several reference points to improve volume alignment during the fusion procedure itself.

In case of redo fusion, the markers are placed in the aorta or its principal branches, with more accurate overlay in terms of aortic spatial orientation. During endovascular aortic repair, the insertion of stiff guides and shafts modify the physiological aortic curvature. Therefore, it is essential to have markers that follow the adaptation of the vessel after the introduction of stiff guides, as in case of redo fusion. In this technique, the placed stent follows the “new” morphology of the vessel. Using the redo fusion technique, which utilizes the stent placed in the initial operation, allows the operator a greater orientation accuracy.

In addition, a high rate of accuracy of the redo fusion has been seen in the thorax; the discrepancy of the bone in the patient position during the pre-operative CTA (upper limbs in hyper-abduction) and the intra-operative position (arms adducted with the elbows extended) is overcome using endovascular markers.

In this context, redo fusion represents a high-precision technique compared with bone fusion, as the markers are not modified by the intra-operative setup. Our study showed that the accuracy was even higher when both the distance between fusion marker and target vessel was lower than 3 cm, and the aortic angle was greater or equal to 45°.

Several pathologies benefiting from this innovative fusion method following a previous operation have been identified, including descending thoracic aortic diseases (TEVAR), thoraco-abdominal aneurysms, type IA and type IB endoleak (F-BEVAR procedure) after EVAR and staged procedures of complex aortic diseases. Thus, redo fusion can be considered a highly refined method and currently represents a significant opportunity in endovascular redo surgery.

There are some limitations to this study. The study was observational, and the sample size was too small to draw strong conclusions. Moreover, advances could be made in image fusion registration workflow and the possible role of a learning curve was not analyzed.

## 5. Conclusions

In case of endovascular redo aortic procedures, a new registration protocol based on two single-frame images overlayed with previously implanted devices can be used. Support in endovascular re-interventions with 2D–3D IF can enable the safe alignment of anatomical structures shown in VR with 2D fluoroscopy, which can be accurately overlapped with landmarks from the previously placed stent graft. This redo fusion technique is accurate and allows the optimization of X-ray working views, supporting the endovascular navigation and vessel catheterization.

## Figures and Tables

**Table 1:** List of the measurements obtained on the 3D workstation.

Variables	Definition
P1	Inferior margin of the target vessel ostium in digital subtraction angiography (DSA)
P2	Inferior margin of the target vessel ostium in bone fusion
P3	Inferior margin of the target vessel ostium in redo fusion
L1	P1–P2 distance
L2	P1–P3 distance
Working area	The new endovascular redo area containing the target vessel
Aortic angulation	Aortic angulation in the working area
Target vessel	The target vessel nearest the previous endovascular stent-graft

**Table 2:** List of patients’ characteristics.

	Previous Endovascular Treatment	Endovascular Redo Treatment	Target Vessel	Distance between Fusion Redo Marker and Target Vessel	Aortic Angulation in the Working Area	L1 Bone Fusion	L2 “Redo” Fusion	Δ
Patient 1	BEVAR	BEVAR	Aortic bifurcation	150 mm	70°	7 mm	1.5 mm	5.5
Patient 2	TEVAR	BEVAR	Celiac artery	50 mm	60°	4 mm	1 mm	3
Patient 3	TEVAR	BEVAR	Celiac artery	25 mm	55°	6 mm	2.5 mm	3.5
Patient 4	FEVAR	EVAR	Right renal artery	5 mm	40°	6 mm	1.5 mm	4.5
Patient 5	BEVAR	BEVAR	Celiac artery	10 mm	20°	5 mm	1.5 mm	3.5
Patient 6	FEVAR	RELINING FEVAR	Left renal artery	5 mm	50°	3 mm	0.3 mm	2.7
Patient 7	EVAR	TEVAR	Celiac artery	50 mm	30°	5 mm	1 mm	4
Patient 8	TEVAR	TEVAR	Left common carotid artery	25 mm	60°	8 mm	1 mm	7
Patient 9	TEVAR	RELINING TEVAR	Celiac artery	50 mm	50°	8 mm	2.5 mm	5.5
Patient 10	TEVAR	FEVAR	Celiac artery	200 mm	65°	6 mm	2.2 mm	3.8
Patient 11	TEVAR	FEVAR	Left common carotid artery	15 mm	70°	7 mm	1 mm	6
Patient 12	TEVAR	FEVAR	Celiac artery	30 mm	60°	6 mm	1 mm	5
Patient 13	TEVAR	TEVAR	Left subclavian artery	15 mm	70°	7 mm	1 mm	6
Patient 14	TEVAR	BEVAR	Celiac artery	60 mm	50°	4 mm	1 mm	3
Patient 15	TEVAR	RELINING TEVAR	Celiac artery	70 mm	10°	3 mm	2 mm	1
Patient 16	FEVAR	RELINING FEVAR	Right renal artery	5 mm	60°	4 mm	0.5 mm	3.5
Patient 17	EVAR	RELINING EVAR	Right renal artery	4 mm	65°	5 mm	1 mm	4
Patient 18	FEVAR	TEVAR	Left renal artery	7 mm	40°	3 mm	1 mm	2
Patient 19	EVAR	RELINING EVAR	Left renal artery	5 mm	70°	7 mm	2.5 mm	4.5
Patient 20	FEVAR	FEVAR	Right renal artery	10 mm	55°	3 mm	1 mm	2

**Table 3:** Variation between intra-observer and interobserver measurement in L1 and L2.

		Bone Fusion	Redo Fusion
Intra-observer measurements	Median distance	0.85 mm	0.81 mm
	Standard error	0.15	0.1
	*p*-value	0.2048	
Interobserver measurement	Median distance	0.71 mm	0.65 mm
	Standard error	0.2	0.05
	*p*-value	0.7952	

**Table 4:** Analysis of accuracy of measurement over 20 patients by paired *t* tests.

	L1	L2	*p*-Value	IC
Median distance	5.350 mm	1.350 mm	<0.0001	3.284–4.716
SD	1.694	0.658		
SEM standard error	0.379	0.147		
Number of patients	20	20		

**Table 5:** Analysis of accuracy of measurement over two patient subgroups.

Distance between Fusion Redo Marker and Target Vessel		L1	L2	*p*-Value	IC
>3 cm	Median distance	5.286 mm	1.600 mm	0.0008	2.231–5.141
	SD	1.799	0.635		
	SEM	0.680	0.240		
	N. of patients	7	7		
≤3 cm	Median distance	5.385 mm	1.215 mm	<0.0001	3.237–5
	SD	1.710	0.654		
	SEM	0.474	0.181		
	N. of patients	13	13		

**Table 6:** Analysis of accuracy of measurement over two patient subgroups.

Angle		L1	L2	*p*-Value	IC
<45°	Median distance	4.400 mm	1.400 mm	0.0100	1.190–4.810
	SD	1.342	0.418		
	SEM	0.600	0.187		
	N. of patients	5	5		
≥45°	Median distance	5.800 mm	1.333 mm	<0.0001	3.705–5.229
	SD	1.568	0.733		
	SEM	0.405	0.189		
	N. of patients	15	15		

## Data Availability

Data sharing is not applicable to this article.
